# Highly Efficient and Stable Iridium Oxygen Evolution Reaction Electrocatalysts Based on Porous Nickel Nanotube Template Enabling Tandem Devices with Solar‐to‐Hydrogen Conversion Efficiency Exceeding 10%

**DOI:** 10.1002/advs.202104938

**Published:** 2022-01-24

**Authors:** Yungi Nam, Daehan Kim, Jinwoo Chu, Na‐Yeon Park, Tae Gun Kim, Kyung Joong Kim, Soo‐Hyun Kim, Byungha Shin

**Affiliations:** ^1^ Department of Materials Science and Engineering Korea Advanced Institute of Science and Technology (KAIST) Daejeon 34141 Republic of Korea; ^2^ School of Materials Science and Engineering Yeungnam University 214‐1, Dae‐dong Gyeongsan‐si 38541 Republic of Korea; ^3^ Surface Analysis Team Korea Research Institute of Standards and Science 267 Gajeong‐ro, Yuseong‐gu Daejeon 34113 Republic of Korea

**Keywords:** facile mass transportation, Ir based OER catalyst, Ir‐Ni synergy, STH over 10%, unassisted water splitting

## Abstract

Ir is one of the most efficient oxygen evolution reaction (OER) catalysts; however, it is also one of the rarest and most expensive elements. Therefore, it is highly desirable to develop Ir catalysts with nanostructures that reduce Ir consumption by maximizing the surface‐to‐volume ratio without limiting the mass transport of reactants and products of reactions. Ir OER catalysts on a template that consisted of porous nanotubes (PNTs) based on Ni are fabricated. The Ir/Ni PNTs offer multiple benefits, including high catalytic performance (potential of 1.500 V vs. reversible hydrogen electrode (RHE) at an operating current density of 10 mA cm^−2^ and Tafel slope of 44.34 mV decade^−1^), minimal use of Ir (mass activity of 3273 A g^−1^ at 1.53 V vs RHE), and facile mass transport through the NT‐sidewall pores (stable operation for more than 10 h). The Ir/Ni PNTs are also applied to a tandem device, consisting of a Cu(In,Ga)Se_2_‐based photocathode and halide perovskite photovoltaic cell, for unassisted water splitting. A solar‐to‐hydrogen conversion efficiency that exceeded 10% is also demonstrated, which is nearly 1% point greater than when a planar Ir film is used as the anode instead of Ir/Ni PNTs.

## Graphical abstract

A highly efficient oxygen evolution reaction catalyst consisting of a small amount of Ir decorated on a vertically aligned Ni porous nanotube template is presented. Thanks to its structural advantages, a potential of 1.500 V versus reversible hydrogen electrode (RHE) at 10 mA cm^−2^ and an ultrahigh mass activity of 3273 A g^−1^ at 1.53 V versus RHE are achieved.

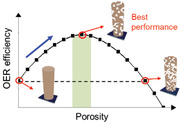

## 1 Introduction

Hydrogen has the advantages of being ecofriendly and abundant and is considered to be an alternative‐energy source that will help replace fossil fuels in the future.^[^ ^1^, ^2^ ^]^ Methods for producing clean hydrogen with solar energy are actively being researched. These methods include photoelectrochemical (PEC) water electrolysis and photovoltaic (PV)‐powered water electrolysis.^[^ ^3^, ^4^, ^5^, ^6^, ^7^, ^8^, ^9^ ^]^ In the PEC system, photoelectrodes and electrocatalysts are both critical components. The hydrogen evolution reaction at the cathode is relatively simple and the reaction kinetics are fast when a proper catalyst such as Pt is used.^[^ ^10^ ^]^ However, the oxygen evolution reaction (OER) is sluggish and requires a relatively large overpotential.^[^ ^11^ ^]^ Thus, the overall water‐splitting reaction is restrained by the OER reaction. This has led to an extensive effort to develop more efficient OER catalysts.^[^ ^12^, ^13^, ^14^, ^15^ ^]^ Ir is one of the best OER catalysts; the Tafel slope and overpotential at the operating current density of 10 mA cm^−2^ of the state‐of‐the‐art Ir catalysts are 49 mV dec^−1^ and 240 mV, respectively.^[^ ^16^ ^]^ However, the rarity and high cost of Ir are ongoing concerns. Therefore, high‐performance Ir OER catalysts that use a minimal amount of Ir continue to be actively pursued.^[^ ^17^, ^18^ ^]^

Nanostructure fabrication is a notable approach for reducing Ir use without losing catalytic activity.^[^ ^19^, ^20^, ^21^, ^22^ ^]^ For example, Tan et al. used IrO*_x_* catalysts in the form of nanoparticles to achieve high catalytic performance with a small amount of Ir: a potential, *E_j_*_=10_, of 1.48 V at an operating current of 10 mA cm^−2^, and a mass activity of 1400 A g_Ir_^−1^.^[^ ^19^ ^]^ In a study by Böhm et al., a matrix consisting of porous antimony‐doped, tin‐oxide microparticles embedded with IrO*_x_* nanoparticles exhibited a potential of 1.47 V at 1 mA cm^−2^, which was ≈50 mV lower than that of the Ir nanoparticle catalyst.^[^ ^20^ ^]^ While an assembly of nanoparticles provides the largest surface area to volume ratio, the entire surface of such a structure may not be fully accessible to reactants during electrochemical reactions due to serpentine diffusion pathways. An array of nanowires (NWs) is another nanostructure that has been widely used as a template for catalyst loading. It is relatively easy to fabricate using a hydrothermal method, for example.^[^ ^23^ ^]^ A nanotube structure is even better for maximizing the surface area (i.e., potential reaction sites) per unit volume because inner surfaces are also accessible.^[^ ^24^ ^]^ However, as the aspect ratio increases, mass transport inside nanotubes becomes increasingly difficult. Therefore, carefully designed nanostructures that maximize the surface‐to‐volume ratio, and thus reduce the use of precious electrocatalysts, without limiting mass transport are highly desirable.

In this work, we have significantly improved the mass activity of Ir OER catalysts using a porous nanotube (PNT) template. The PNT structure maximizes the surface area (both the inner and outer walls of the nanotubes are utilized) and enhances the mass transport of the OER reactants and products via pores through the sidewalls of the nanotubes (NTs). PNTs were prepared by chemically etching Ni‐coated, vertically aligned ZnO NWs. ZnO was etched much faster than Ni in an aqueous HCl solution, which led to the complete removal of the ZnO while keeping the Ni overlayers in the form of NTs with pores. Finally, Ir catalysts were deposited onto the Ni PNTs by electroplating or atomic layer deposition (ALD). The ultrahigh mass activity of 3273 A g^−1^ at 1.53 V versus reversible hydrogen electrode (RHE) and an *E_j_*_=10_ of 1.500 V versus RHE with a Tafel slope of 44.34 mV decade^−1^ in 1 m of KOH electrolyte (pH = 13.5) were achieved with the PNT structure. We have chosen a potential at the operating current density of 10 mA cm^−2^ as a parameter to gauge the (photo)electrochemical performance. However, one should note that the level of operating current densities in industrial water electrolysis, which are on the order of 1–2 A cm^−2^, much larger than 10 mA cm^−2^. The high catalytic performance of our nanostructured Ir was further applied to a PEC‐PV tandem device consisting of a Cu(In,Ga)Se_2_ (CIGS)‐based photocathode and a halide perovskite solar cell for unassisted water splitting. During a comparison test with a reference device (Ir film OER catalysts with the same quality CIGS and perovskite), the Ir PNT catalysts improved the solar‐to‐hydrogen (STH) conversion efficiency from 9.92% to 10.86%, which illustrates the importance of high‐performance Ir catalysts in PEC water electrolysis.

## 2 Results and Discussion

**Figure 1:**
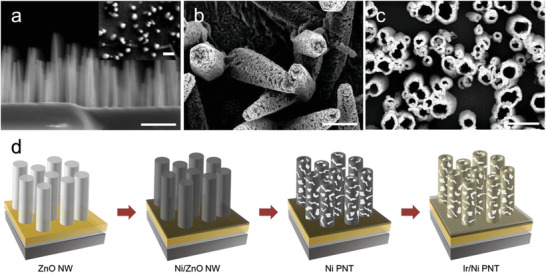
SEM images of a) ZnO NWs (inset: top view), b) Ni‐coated NWs, and c) Ni PNTs. Scale bars are 2 µm. d) Schematic illustration of the formation of Ir/Ni PNTs.

**Figure** **1** illustrates the process by which Ir‐coated Ni PNTs were formed. First, vertically aligned ZnO NWs were synthesized via a hydrothermal method.^[^ ^23^ ^]^ A 100 nm thick Au layer was deposited on a Si (111) wafer to reduce the lattice mismatch between the Si (111) plane and the ZnO nanowires and a thin layer of Cr was used between the Au and Si wafer as an adhesion layer.^[^ ^25^ ^]^ The crystallinity of the surface onto which ZnO NWs are grown is known to be crucial for the alignment of the NWs; therefore, the Au/Cr/Si substrate was annealed at 300 °C for 40 min. The formation of well‐aligned ZnO NWs with a diameter of ≈300 nm and a height in the range of 3–5 µm was confirmed by scanning electron microscopy (SEM) images, as shown in Figure 1a. Ni was conformally deposited on the ZnO NWs by electrodeposition (Figure 1b). The Ni‐coated ZnO NWs were then subjected to HCl etching. ZnO in a 10 vol% aqueous HCl solution is etched much faster than Ni, and in less than 10 s of etching, the inner ZnO nanowires were almost completely removed, while the outer Ni layer was partially etched. The etching left pores in the walls of the hollow Ni nanotubes. These pores are clearly visible in the SEM images shown in Figure S1 in the Supporting Information. At this point, the Ni PNTs served as a template for the Ir coating (Figure 1c). The formation of crystalline Ni layers and the removal of ZnO after the etching step were confirmed by X‐ray diffraction (XRD) patterns (Figure S2a, Supporting Information) and SEM energy dispersive X‐ray spectrometry (EDS) elemental analysis (Figure S2c, Supporting Information) of the Ni PNTs. Differing amounts of Ir were electrodeposited onto the Ni PNT templates using a 5 × 10^−3^ m H_2_IrCl_6_ solution at 0.4 V by varying the deposition duration (0.5, 2, 5, and 10 h). These samples are hereafter referred to as “ED‐Ir/Ni PNTs.” Figure 1d presents a schematic illustration of the synthesis of the Ir/Ni PNT catalysts. No discernable peak corresponding to Ir was observed in the XRD analysis of the Ir/Ni PNTs (Figure S2a, Supporting Information) because the amount of Ir deposited was not sufficient to be clearly detected by XRD. The atomic% of Ir for the ED‐Ir/Ni PNTs determined by SEM EDS was in the range of 4–5% (Figure S2d, Supporting Information). The comparison of a bare Ir film and an Ir‐coated Ni film (the Ir thickness ≈4 nm) confirms the formation of Ir‐Ni alloy when the two are in contact, as evidenced by the shift of the Ir 4f peaks to higher binding energies (Figure S3, Supporting Information).^[^ ^26^ ^]^

**Figure 2:**
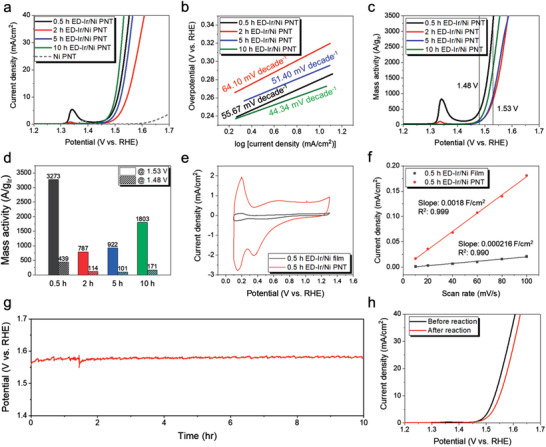
a) LSV curves and b) Tafel plots from the polarization curves of ED‐Ir/Ni PNTs with varying Ir contents. c) LSV curves normalized by the Ir mass and d) mass activities of the Ir electrocatalysts measured at 1.53 V (solid color) and 1.48 V (hatched pattern) versus RHE. e) CV curves of Ir/Ni film and Ir/Ni PNT at a scan rate of 20 mV s^−1^. f) Double‐layer capacitance of Ir/Ni film and PNTs determined from the slopes of current density versus scan rate (from 10 to 100 mV s^−1^). Current density was measured at 1.15 V versus RHE. g) Chronopotentiometric measurement of the OER by ED‐Ir/Ni PNTs and h) LSV curves of ED‐Ir/Ni PNTs before and after 10 h of OER . All electrochemical measurements were performed in 1 m KOH.

The catalytic activity of the ED‐Ir/Ni PNTs with different Ir‐loading amounts was studied. **Figure** **2**a,b presents iR‐corrected dark linear sweep voltammetry (LSV) curves measured in a 1 m KOH solution and the corresponding Tafel slopes of the ED‐Ir/Ni PNTs with different durations of Ir electroplating. The raw LSV curves before iR correction are shown in Figure S4 in the Supporting Information. For Ir deposition times of 2 h or longer (up to 10 h), both *E_j_*_=10_ and the Tafel slope decrease, that is, there is a smaller reaction barrier and faster OER kinetics with increasing mass of Ir. However, the electrodeposition of Ir for more than 10 h was not possible because the electroplating solution (5 × 10^−3^ m H_2_IrCl_6_ solution) was strongly acidic (pH ≈ 1.7) and caused the dissolution of the Ni PNT template when processing durations were longer than 10 h. The 10 h ED‐Ir/Ni PNT catalyst showed the best performance: *E_j_*_=10_ of 1.500 V versus RHE and a Tafel slope of 44.34 mV decade^−1^, both of which are comparable to those of state‐of‐the‐art Ir OER catalysts reported in the literature. Notably, the *E_j_*_=10_ value of 1.500 V was the lowest among the Ir OER catalysts used in an alkaline electrolyte (**Table** **1**). In general, the overpotential of a high‐performance Ir OER catalyst operating in an alkaline electrolyte is less than that in an acidic electrolyte by as much as 20 mV.^[^ ^27^ ^]^ The electrochemical measurements in this study were carried out in basic electrolytes because our Ir/Ni PNTs have poor stability in an acidic medium due to the dissolution of Ni. However, with the application of a proper protection layer, we expect that the Ir/Ni PNTs would achieve an even better (lower) *E_j_*_=10_ value when used in an acidic electrolyte.

**Table 1:** Potentials at 10 mA cm^−2^, mass activities, and Tafel slopes of Ir‐based catalysts

	Structure	*E* at 10 mA cm^−2^ (*E_j_*_=10_)[v]	Mass activity [A g^−1^]	Tafel slope [mV dec^−1^]	Electrolyte	Year	Ref.
Ir‐Ni	Porous nanowire (ED 10 h)	1.500	1803 (@1.53 V)	44.34	Alkaline 1 m KOH	2021	This work
Ir‐Ni	Porous nanowire (ED 0.5 h)	1.510	3273 (@1.53 V)	55.67	Alkaline 1 m KOH	2021	This work
Ir	Ir@vertically grown graphene	1.55	–	52	Alkaline 1 m KOH	2019	^[^ ^29^ ^]^
Ir	Ir@Pt sphere	1.485	–	65	Acidic 0.1 m HClO_4_	2018	^[^ ^30^ ^]^
Ir	Film	1.523	39 (@ 1.525 V)	45	Acidic 0.05 m H_2_SO_4_	2019	^[^ ^31^ ^]^
Ir‐Ni	Film	1.501	410 (@ 1.53 V)	35	Acidic 0.05 m H_2_SO_4_	2019	^[^ ^31^ ^]^
Ir‐Ni	Bulk	1.53	325 (@ 1.53 V)	–	Acidic 0.1 m HClO_4_	2015	^[^ ^17^ ^]^
IrO*_x_*	Ir@TKK nanoparticle	1.48	1400 (@1.53 V)	49	Acidic 0.5 m H_2_SO_4_	2019	^[^ ^19^ ^]^
SrIrO_3_	6H‐SrIrO_3_	1.478	75 (@ 1.525 V)	–	Acidic 0.5 m H_2_SO_4_	2018	^[^ ^18^ ^]^
SrIrO_3_	3C‐SrIrO_3_	1.5	40 (@ 1.525 V)	–	Acidic 0.5 m H_2_SO_4_	2018	^[^ ^18^ ^]^

The oxidation peak at ≈1.35 V that is present in some of the LSV curves in Figure 2a was due to the oxidation of Ni from the 2+ to the 3+ valence state.^[^ ^28^ ^]^ The Ni oxidation peak became weaker at an extended Ir‐deposition time and disappeared completely when the deposition time was equal to or greater than 5 h. Given the disappearance of the Ni oxidation peak, we inferred that the contribution of the underlying Ni and Ir‐Ni alloy to the catalyst activity would diminish with sufficient Ir (electrodeposited for 5 h or longer). Moreover, the 0.5 h ED‐Ir/Ni PNT catalyst yielded an even lower *E_j_*_=10_ than the sample that underwent electrodeposition of Ir for 5 h, although the Ir content was nearly ten times lower, assuming a nearly constant growth rate of Ir during the electrodeposition. We attributed the improved catalytic performance of the 0.5 h ED‐Ir/Ni PNT sample to the coexistence of Ir and Ni near the surface region. As discussed later, the synergistic effect of Ir and Ni in improving OER catalysts was studied further, using a model structure. This model structure was a set of Ir films with varying coverage and thickness on Ni substrates, which made it easier to measure the exact quantity of Ir.

The mass of Ir in our Ir/Ni PNTs was measured by inductively coupled plasma mass spectrometry, as listed in Table S1 in the Supporting Information, and the mass activities were calculated (Figure 2c,d). For a direct comparison with state‐of‐the‐art Ir catalysts reported in the literature, the mass activities of our samples were evaluated at both 1.48 and 1.53 V versus RHE (see Table 1). Given the large surface area and enhanced mass transport of our catalyst design, all samples exhibited high mass activity that exceeded 700 A g^−1^ at 1.53 V (Figure 2d). In particular, the 10 h ED‐Ir/Ni PNTs, the sample with the highest catalytic performance, showed mass activities of ≈1803 A g^−1^ at 1.53 V and 171 A g^−1^ at 1.48 V, while the 0.5 h ED‐Ir/Ni PNTs, which had the lowest loading of Ir, showed the highest mass activities of 3273 A g^−1^ at 1.53 V and 439 A g^−1^ at 1.48 V. The excellent OER performance of the Ir/Ni PNT catalyst originates from its large surface area and facile mass‐transport ability. Cyclic voltammetry (CV) curves of the 0.5 h‐ED Ir/Ni film and the 0.5 h ED‐Ir/Ni PNT catalysts are shown in Figure 2e. The CV curves were measured in a potential range of 0.1–1.3 V versus RHE. The area under the CV curve for the 0.5 h‐ED Ir/Ni PNTs is much larger than that for the 0.5 h‐ED Ir/Ni film catalyst, which indicates that there are a larger number of active sites on the surface (i.e., larger surface area) of the PNTs. The measurement of the surface areas per unit projected‐sample area of both samples was conducted by electrochemical surface area (ECSA), which can be estimated by the double‐layer capacitance (*C*_dl_) (Figure 2f). The *C*_dl_ was determined from the CV curves acquired with various scan rates in a potential range between 1.05 and 1.25 V. The use of this range avoided a Faradaic potential region where electron transfer causes oxidation or reduction to occur (Figure S5, Supporting Information). The ECSA of 0.5 h ED‐Ir/Ni PNTs is larger than that of 0.5 h ED Ir/Ni film by almost an order of magnitude. The surface area ratio between the film and the PNT structure was also confirmed by a simple geometrical consideration, and based on a representative SEM image (Figure S6a, Supporting Information), we identified that the areal density of nanotubes is ≈7.6 × 10^11^ nanotubes cm^−2^ and each nanotube roughly measures ≈0.5 µm in the outer diameter, ≈0.4 µm in the inner diameter, and ≈5 µm in the height, which together yields a total surface area per unit projected area of 1 cm^2^—10.7, that is, ≈11 times as large as a planar structure, which is consistent with the estimation by ECSA (Figure S6, Supporting Information).

The stability of the Ir/Ni PNT catalysts was tested via continuous chronopotentiometric measurements at an operating current density of 10 mA cm^−2^ (Figure 2g). For the stability test, we deliberately chose the sample that we expected to be the most unstable and not the best‐performing among our PNT catalysts. The chosen sample was the 0.5 h ED‐Ni/Ir PNTs, which had the lowest Ir loading, that is, the largest chance of exposure of Ni, and thus was more susceptible to degradation in the liquid electrolyte. As shown in Figure 2g, stable operation continued for more than 10 h. The LSV curves of the 0.5 h ED‐Ir/Ni PNTs before and after 10 h of continuous OER reaction at 10 mA cm^−2^ are compared in Figure 2h. The value of *E_j_*_=10_ slightly increased from 1.56 V at the beginning to 1.58 V after the reaction; the Tafel slope also slightly increased after the reaction. The comparison of the X‐ray photoelectron spectroscopy (XPS) spectra of Ir 4f region before and after 10 h of OER reaction shown in Figure S7 in the Supporting Information verifies that some dissolution of Ir, as evident from the reduced XPS peak intensity, which must be responsible for the initial performance degradation in Figure 2g. The ratio of Ir^3+^ (from Ir(OH)_3_ or Ir_2_O_3_) and Ir^4+^ (from IrO_2_) to Ir^0^ increases after the reaction, indicating that the metallic Ir converts to Ir (hydr)oxides with higher stability during the reaction. SEM images taken after 10 h of reaction revealed that there was no noticeable structural damage to the Ir/Ni PNTs (Figure S8, Supporting Information).

**Figure 3:**
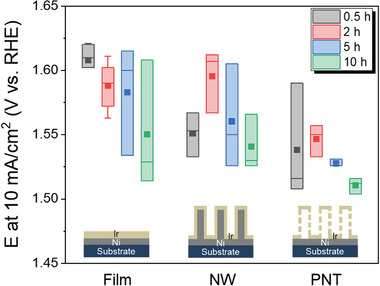
Potentials at 10 mA cm^−2^ for the ED‐Ir films, NWs, and PNTs with different Ir electrodeposition times.

The superior performance of our Ir catalyst design was confirmed by direct comparisons with other structures, specifically a planar film and NWs (**Figure** **3** and Figure S9, Supporting Information). Figure 3 presents the values of *E_j_*_=10_ for the ED‐Ir/Ni film, ED‐Ir/Ni NW, and ED‐Ir/Ni PNT catalysts. Schematics of the different catalyst structures are shown at the bottom of Figure 3. The same set of electrodeposition times of Ir (0.5, 2, 5, and 10 h) was used for all three structures including PNTs; therefore, the loaded amounts of Ir can be considered similar between the different structures. The lowest *E_j_*_=10_ of the PNTs was immediately noticeable compared to the others for a given loading of Ir. Considering that similar amounts of Ir were used across the different structures, our results highlight the importance of careful design of the catalyst structure. The performance of the films was the worst, as anticipated, and the trend of *E_j_*_=10_ versus the Ir loading amounts of the NWs was similar to that of the PNTs: the lowest overpotential came from the highest Ir loading (10 h) and the second best came from the lowest loading level (0.5 h), not from the second highest Ir loading (5 h). The structural advantages of the PNTs were expanded to Ir catalysts grown by ALD. Ir films with a thickness of 2 nm were prepared by ALD on planar Ni films, Ni NWs, and Ni PNT templates. To improve statistical validity, two batches of samples were prepared and tested. As shown in Figure S10 in the Supporting Information, the *E_j_*_=10_ results were in the following order: ALD‐Ir/Ni PNTs < ALD‐Ir/Ni NWs < ALD‐Ir/Ni films. This demonstrates that our PNT structure improved catalytic activity due to the larger number of reaction sites.

**Figure 4:**
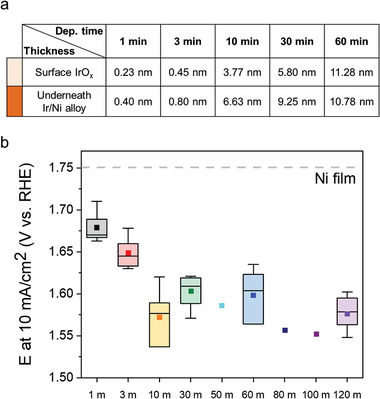
a) The thicknesses of surface IrO*_x_* and underneath Ir‐Ni alloys of ED‐Ir/Ni films with different Ir electrodeposition times, as determined by MEIS measurements. b) *E_j_*_=10_ of ED‐Ir/Ni films. The dashed line at ≈1.75 V represents the result from a Ni film without Ir. Data points for the 50, 80, and 100 m were from a single batch while the other points were from multiple (more than five) batches.

The synergy between Ir and the Ni host was further studied using ED‐Ir/Ni films with various amounts of Ir. Compositional information about the samples, including the thicknesses of Ir, was obtained by medium‐energy ion scattering (MEIS), as shown in Figure S11 in the Supporting Information and **Figure** **4**a. For ED times up to 3 min, the position of the Ni edge is almost the same as that of the pure Ni film, indicating that Ni is exposed on the surface. Thus, there was not full Ir coverage (Figure S11a, Supporting Information). For samples with full Ir coverage, multiple layers consisting of Ni‐Ir alloys with gradually varying Ni/Ir ratios and surface IrO*_x_* were assumed to fit the MEIS spectra (Figure S11b,c, Supporting Information). A plot of *E_j_*_=10_ from one batch (for the 50, 80, and 100 m data points) or five or larger batches (for all the other data points) of the ED‐Ir/Ni films with various Ir contents are shown in Figure 4. The performance initially improved until full coverage of Ir at an ED time of 10 min, then degraded from 10 to 50 min of ED. After ED times greater than 50 min, the performance improved again. The most active catalyst structure is expected to be was thick, bulk‐like Ir, which is a more efficient catalyst than Ni. Alternatively, considering that the coexistence of Ir and Ni has been proposed to help lower the catalytic reaction barrier,^[^ ^26^ ^]^ multilayers with surfaces that are fully covered with the thinnest possible layer of Ir on Ni (or Ni‐Ir alloy) would also be efficient, although achieving complete coverage, with no exposed Ni on the surface, with a thin layer of Ir would not be trivial. Considering the MEIS and electrochemical results, the 10 min ED Ir‐Ni film was the most efficient multilayer structure among the samples.

**Figure 5:**
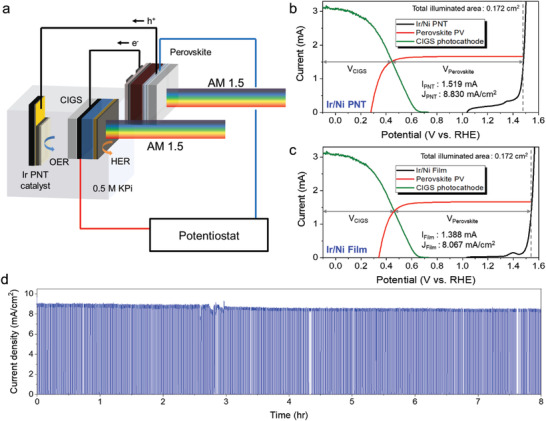
a) Schematic illustration of a tandem CIGS photocathode‐perovskite PV‐Ir/Ni PNTs anode for unassisted water splitting under a parallel illumination configuration. Overlaid photocurrent‐potential curves of the components of the tandem PEC‐PV cell and the b) Ir/Ni PNTs and c) Ir film under parallel light illumination. Gray vertical dashed lines are the short‐circuit condition of the perovskite. d) Chronoamperometry measurement of a two‐terminal CIGS‐perovskite‐Ir PNTs anode at zero applied bias under one‐sun illumination. A 0.5 m KPi (pH 6.58) solution was used as the working electrolyte.

The excellent OER catalytic properties of the Ir/Ni PNTs were utilized in PEC‐PV tandem devices for unassisted (unbiased) water splitting. The tandem device consisted of a CIGS photocathode, a halide perovskite PV, and an Ir anode. We previously improved the PEC performance and stability of CIGS photocathodes by introducing ZnS/CdS buffer layers^[^ ^32^ ^]^ and a reduced graphene oxide catalyst binder.^[^ ^33^ ^]^ These improvements were also applied in the current study. For unassisted water splitting, the sum of the output voltages of the two photovoltage‐producing components in a tandem cell must be sufficiently higher than the thermodynamic requirement, i.e., 1.23 V. Therefore, we used perovskite PV with a wide bandgap (≈1.68 eV) absorber, as opposed to one with a conventional bandgap (≈1.55–1.6 eV). An absorber with a conventional bandgap is more commonly used in high‐efficiency single‐junction perovskite solar cells. In our previous study, we considerably extended the stability and improved the power‐conversion efficiency of wide‐bandgap perovskite solar cells using anion‐engineered 2D passivation layer additives.^[^ ^34^ ^]^ The most common configuration of two light absorbers in a tandem device is vertical stacking, where the two layers are connected electrically and optically in series. However, in our previous study of PEC‐PV tandem devices using CIGS and perovskite of quality levels similar to those in the current study, we found that another configuration, electrically in series but optically in parallel, led to better performance. In this parallel illumination configuration, the CIGS photocathode receives full one‐sun illumination, as opposed to the light filtered by the perovskite. This led to a higher operating current density despite the fact that the total active (illuminated) area became twice as large as that in the series illumination; that is, the total photocurrent from the parallel illumination was larger than twice the photocurrent from the optically series configuration.^[^ ^33^ ^]^ Therefore, in the current study, we chose a parallel illumination configuration, the schematic of which is shown in **Figure** **5**a.

The performance of a tandem device can be estimated by overlaying the light current–potential (*I*–*V*) curves of the two light absorbers and identifying the photocurrent at the point where the two *I*–*V* curves cross, as shown in Figure 5b,c. The structure and individual *I*–*V* curves of the CIGS photocathode and perovskite cell are shown in Figures S12 and S13 in the Supporting Information, respectively. The predicted operating current density was 8.83 mA cm^−2^ (Figure 5b), and the actual two‐terminal chronoamperometric measurement with no applied bias under chopped one‐sun illumination yielded an initial current density of 8.79 mA cm^−2^ (Figure 5d), which was similar to the predicted value. This corresponds to an STH conversion efficiency of 10.81%, assuming 100% Faradaic efficiency (the equation for calculating the STH conversion efficiency and the justification for the 100% Faradaic‐efficiency assumption are shown in Figure S14, Supporting Information). The stability of the device was also excellent; more than 90% of the initial current density was maintained after 8 h of continuous water splitting. The contribution of our nanostructured Ir catalysts to the high STH efficiency was confirmed by comparison with the case where a planar Ir film was used instead of Ir/Ni PNTs. Figure 5c shows the predicted operating current density when the Ir film catalyst with the highest performance was used, as shown in Figure 4. The comparison of the two cases indicates an increase of almost 1% point in the STH conversion efficiency when the Ir film OER catalyst was replaced with the Ir/Ni PNTs, while the performance of the CIGS photoelectrode and perovskite PV remained the same. This illustrates the importance of maximizing the performance of PEC‐cell electrocatalysts to achieve higher STH conversion efficiencies.

## 3 Conclusion

**Figure 6:**
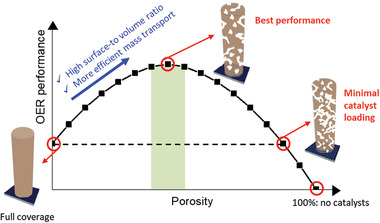
Tunability between catalytic performance and Ir usage in PNT catalysts

We successfully fabricated a PNT template for Ir OER catalysts using the following steps: hydrothermal growth of ZnO NWs, deposition of Ni overlayers, wet‐chemical etching to form hollow Ni NTs with pores in the sidewalls, and finally the decoration of Ir by electrodeposition or ALD. As opposed to commonly used nanostructures for electrocatalysts consisting of precious metal(s), such as NWs and NTs, our PNTs maximize the surface area (both the inner and outer walls of nanotubes were utilized) and also allow the facile transport of reactants and products via the pores in the sidewalls of the nanotubes. By controlling the loading amounts of Ir on the Ni PNT template, the preservation of the expensive raw material can be maximized (with an ultrahigh mass activity of 3273 A g^−1^ at 1.53 V vs RHE); or alternatively, the catalytic performance can be maximized (with an *E_j_*_=10_ of 1.500 V vs RHE and a Tafel slope of 44.34 mV decade^−1^). In particular, when Ir loading was minimal, synergistic effects from the coexistence of Ir and Ni near the surface appeared to improve the overall catalytic activity. Further tunability of our PNT structure can be envisioned, which would balance material cost savings with performance. As shown in **Figure** **6** and Figure S15 in the Supporting Information, the porosity of NTs can be controlled by adjusting the wet‐etching duration, which creates a continuum between the best catalytic performance and the minimal usage of the catalysts. Our PNT structure is also universally applicable to materials other than Ni and Ir. PNTs can be fabricated with any metal, as long as the metal is etched slower than ZnO in an aqueous acidic solution. These metal PNTs could then be decorated with any material that has catalytic activity.

The high catalytic performance of our nanostructured Ir was applied to a PEC‐PV tandem device consisting of a CIGS‐based photocathode and a halide perovskite for unassisted water splitting. In a comparison test with a control device consisting of Ir film OER catalysts with CIGS and perovskite of the same quality, when the anode was changed to Ir PNTs, the STH conversion efficiency increased from 9.92% to 10.86%. This illustrates the importance of high‐performance Ir catalysts in PEC water electrolysis.

## 4 Experimental Section

### Synthesis of ZnO Nanowire

Si (111) wafers were cleaned with acetone, ethanol, and deionized (DI) water, and 20 nm thick Cr and 100 nm thick Au layers were sequentially deposited using an e‐beam evaporator. Postdeposition heat treatment was conducted at 300 °C for 40 min in air. ZnO nanowires were synthesized on the heat‐treated Si/Cr/Au wafers by a hydrothermal method using a solution consisting of 10 mmol of Zn(NO_3_)_2_∙6H_2_O (Sigma‐Aldrich, reagent grade, 98%) and 10 mmol of hexamethylenetetramine (Sigma‐Aldrich, American Chemical Society (ACS) reagent, ≥99.0%) in 500 mL of DI water. The precursor solution was stirred overnight before use. The precursor solution (30 mL) was added to a 100 mL Teflon‐lined stainless‐steel autoclave with a Si/Cr/Au substrate placed facing down on top of the precursor solution. The autoclave was sealed and kept at 90 °C for 12 h and cooled naturally outside the oven for 1 h.

### Synthesis of Porous Ni Nanotubes

The electrolyte used for the electrodeposition of Ni was a mixture of 0.05 mol of NiSO_4_∙6H_2_O (Sigma‐Aldrich, ACS reagent, ≥98%), 0.01 mol of NiCl_2_ (Sigma‐Aldrich, anhydrous, powder, 99.99% trace metals basis), and 0.05 mol of H_3_BO_3_ (Sigma‐Aldrich, ACS reagent, ≥99.5%) dissolved in 500 mL of DI water and stirred for 4 h. For each deposition, 40 mL of the Ni electrolyte was used with a Pt coil and Ag/AgCl electrode as the counter electrode and reference electrode, respectively. A fixed area of a substrate was immersed in the solution, onto which a Ni layer was conformally electrodeposited at a current of 0.25 mA for 20 min. After electroplating, the samples were immersed in a 10 vol% diluted HCl solution (≈1.65 m) for 10 s to dissolve the ZnO cores and to create holes on the remaining Ni outer layer. The porosity of the hollow Ni nanotubes can be controlled by varying the etching duration. The Si/Cr/Au/Ni PNT structure was immersed in DI water and carefully washed for 10 s, followed by heat treatment in a vacuum at 100 °C for 10 min.

### Ir Deposition on Ni PNTs

Ir was electroplated on a Ni PNT matrix using an electrolyte consisting of 20 mL of 5 × 10^−3^ m H_2_IrCl_6_∙*x*H_2_O (Sigma‐Aldrich, 99.95% trace metals basis). A Pt coil and Ag/AgCl electrode were used as the counter and reference electrodes, respectively. The electrolyte temperature was maintained at 70 °C and the bias was at 0.4 V. A series of samples with different electrodeposition times (0.5, 2, 5, and 10 h) were prepared for analysis.

ALD was also used as an alternative method to prepare Ir catalysts. Ir thin films were deposited at 250 °C in a travelling‐wave type ALD reactor (NCD, Lucida D100, Korea) using a proprietary Ir metal‐organic precursor, synthesized by Tanaka Precious Metals, Japan. The canister temperature for the Ir precursor was set at 55 °C to provide sufficient precursor for the deposition. O_2_ (purity: 99.9999%) was used as a counter reactant to deposit the Ir thin films. 100 sccm (standard cubic centimeter per minute) of N_2_ was used as the carrier and purge gas for the metal precursors during the deposition process. One cycle of the Ir ALD process consisted of the following steps: precursor pulsing for 7 s, reactant pulsing for 5 s, and purging for 10 s. This condition was established according to self‐limiting growth conditions. The thickness of Ir was controlled by adjusting the number of ALD cycles.

### Statistical Analysis

The rectangles, the filled square dots, and the thin horizontal lines inside each box in Figures 3 and 4 represent the interquartile range, the mean potential, and the median potential, respectively. For the statistical evaluation presented in Figure 3, total 36 samples (3 samples times 12 different configurations, i.e., different Ir deposition time and types of structures) were used. In Figure 4, total 29 samples (one sample for 50, 80, and 100 m and five or six samples for the remaining data points) were used. The results of all measurements used to construct plots in Figures 3 and 4 are shown in Tables S2 and S3 in the Supporting Information.

## Conflict of Interest

The authors declare no conflict of interest.

## Supporting information

Supporting Information

## Data Availability

The data that support the findings of this study are available in the Supporting Information of this article.
