# Supplementary material for: Highly Efficient and Stable Iridium Oxygen Evolution Reaction Electrocatalysts Based on Porous Nickel Nanotube Template Enabling Tandem Devices with Solar‐to‐Hydrogen Conversion Efficiency Exceeding 10%
Source: Adv Sci (Weinh). 2022 Jan 24;9(9):2104938. doi: 10.1002/advs.202104938 (PMC8948658; doi:10.1002/advs.202104938)
Supplement: Supplementary file 1 — Supporting Information [file ADVS-9-2104938-s001.pdf]

ADVANCED SCIENCE
Open Access

Supporting Information

for *Adv. Sci.*, DOI 10.1002/advs.202104938

Highly Efficient and Stable Iridium Oxygen Evolution Reaction Electrocatalysts Based on Porous Nickel Nanotube Template Enabling Tandem Devices with Solar-to-Hydrogen Conversion Efficiency Exceeding 10%

Yungi Nam, Daehan Kim, Jinwoo Chu, Na-Yeon Park, Tae Gun Kim, Kyung Joong Kim, Soo-Hyun Kim and Byungha Shin**

ADVANCED SCIENCE
Open Access

Supporting Information

for *Adv. Sci.,* DOI: 10.1002/advs.202104938

Highly Efficient and Stable Iridium Oxygen Evolution Reaction Electrocatalysts Based on Porous Nickel Nanotube Template Enabling Tandem Devices with Solar-to-Hydrogen Conversion Efficiency Exceeding 10%

Yungi Nam[1], Daehan Kim[1], Jinwoo Chu[1], Na-Yeon Park[2], Tae Gun Kim[3], Kyung Joong Kim[3], Soo-Hyun Kim[2,*], Byungha Shin[1,*]

Supporting Information

Highly Efficient and Stable Iridium Oxygen Evolution Reaction Electrocatalysts Based on Porous Nickel Nanotube Template Enabling Tandem Devices with Solar-to-Hydrogen Conversion Efficiency Exceeding 10%

Yungi Nam[1], Daehan Kim[1], Jinwoo Chu[1], Na-Yeon Park[2], Tae Gun Kim[3], Kyung Joong Kim[3], Soo-Hyun Kim[2,*], Byungha Shin[1,*]

[1] Department of Materials Science and Engineering, Korea Advanced Institute of Science and Technology (KAIST), Daejeon 34141, Republic of Korea.

[2] School of Materials Science and Engineering, Yeungnam University, 214-1, Dae-dong, Gyeongsan-si 38541, Republic of Korea

[3] Surface Analysis Team, Korea Research Institute of Science and Standards, Daejeon, Korea

[*] Corresponding author(s). Email: byungha@kaist.ac.kr, soohyun@ynu.ac.kr

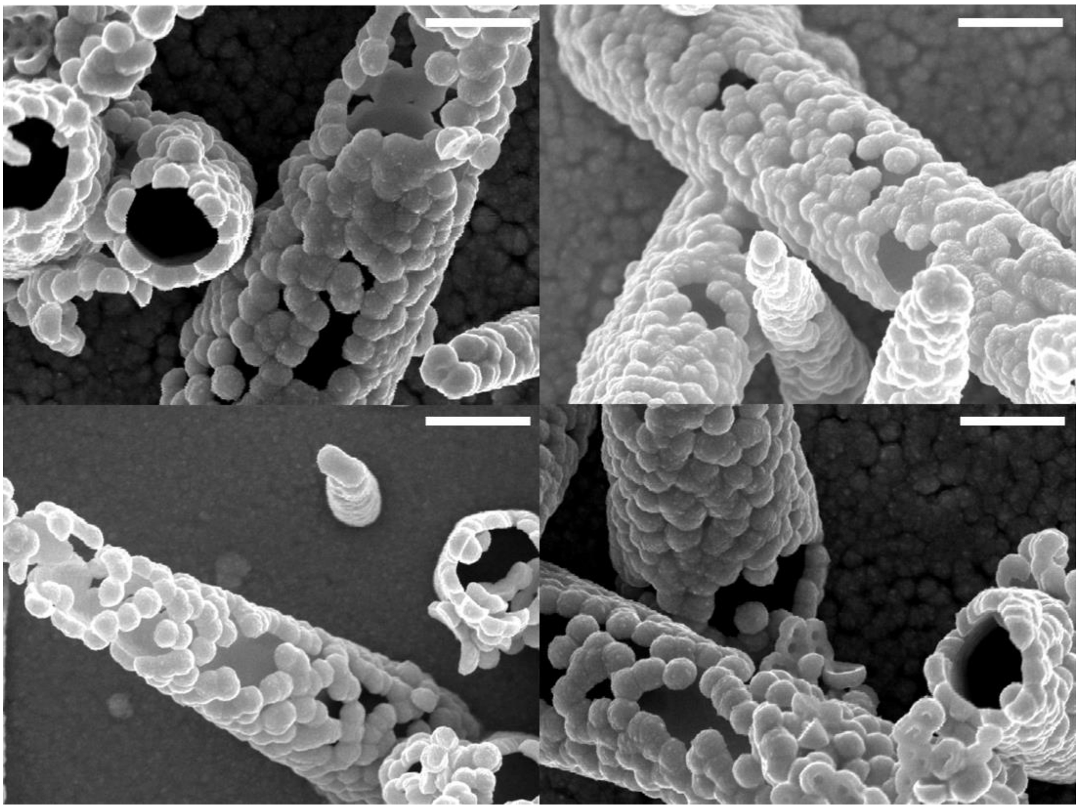

Figure S1. SEM images of Ni PNTs formed after HCl etching. Scale bars are 1 μm.

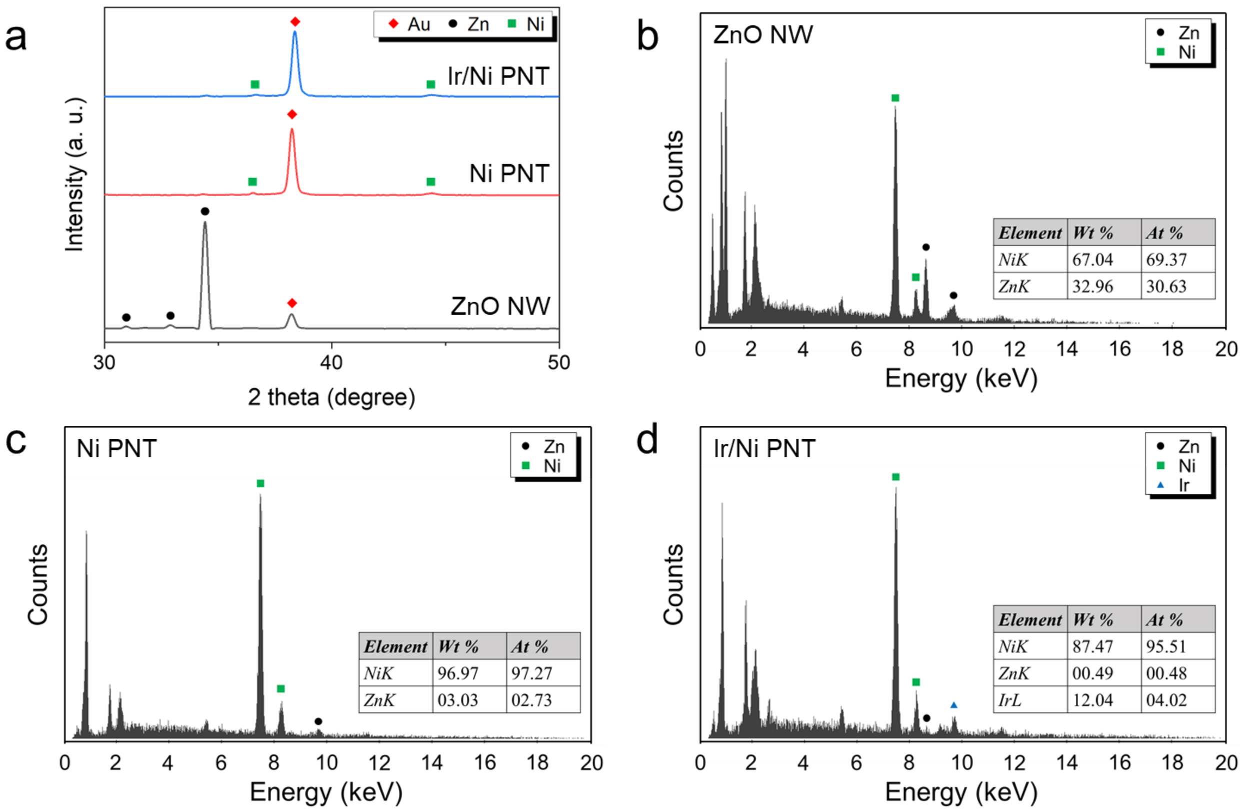

Figure S2. a) XRD patterns of ZnO NWs, Ni PNTs and Ir/Ni PNTs. EDS elemental analysis of b) Ni NWs, c) Ni PNTs, and d) Ir/Ni PNTs. Electrodeposition of Ir was carried out for 10 hours.

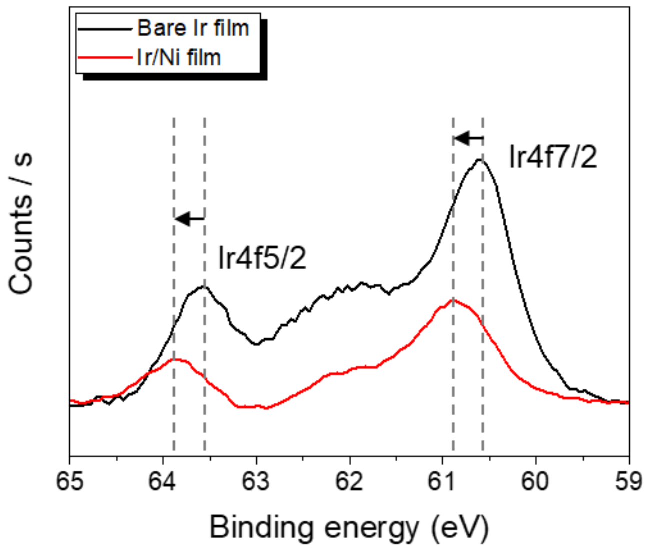

Figure S3. XPS Ir4f spectrum of bare Ir film and Ir/Ni film.

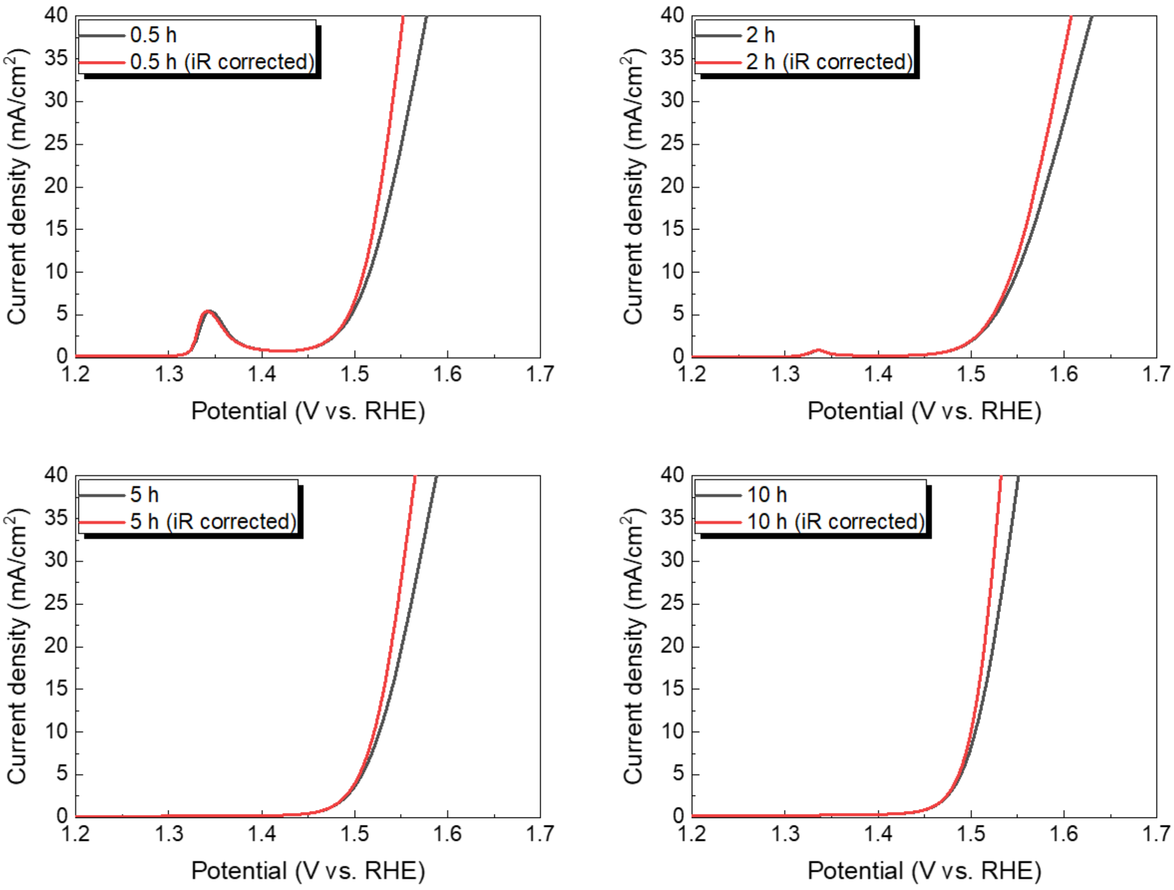

Figure S4. LSV curves of 0.5 h, 2 h, 5 h, 10 h ED-Ir/Ni PNT catalysts before and after the iR correction.

Sample	0.5 h ED-Ir/Ni PNT	2 h ED-Ir/Ni PNT	5 h ED-Ir/Ni PNT	10 h ED-Ir/Ni PNT
Mass of Ir per unit mass of the samples (mg/kg)	47.121	41.993	140.551	99.34
Mass of samples (g)	0.085	0.0775	0.0971	0.0761
Mass of Ir in samples (mg)	0.0040	0.0032	0.0136	0.0076
Area of sample (cm^2)	0.608	0.406	0.882	0.366
Mass of Ir per unit area of the samples (mg/cm^2)	0.0066	0.0080	0.0155	0.0207

Table S1. Mass of Ir in ED-Ir/Ni PNT samples measured by ICP-MS. Masses of Ir in mg/g were converted to mg/cm^2 using the masses per unit area of the samples.

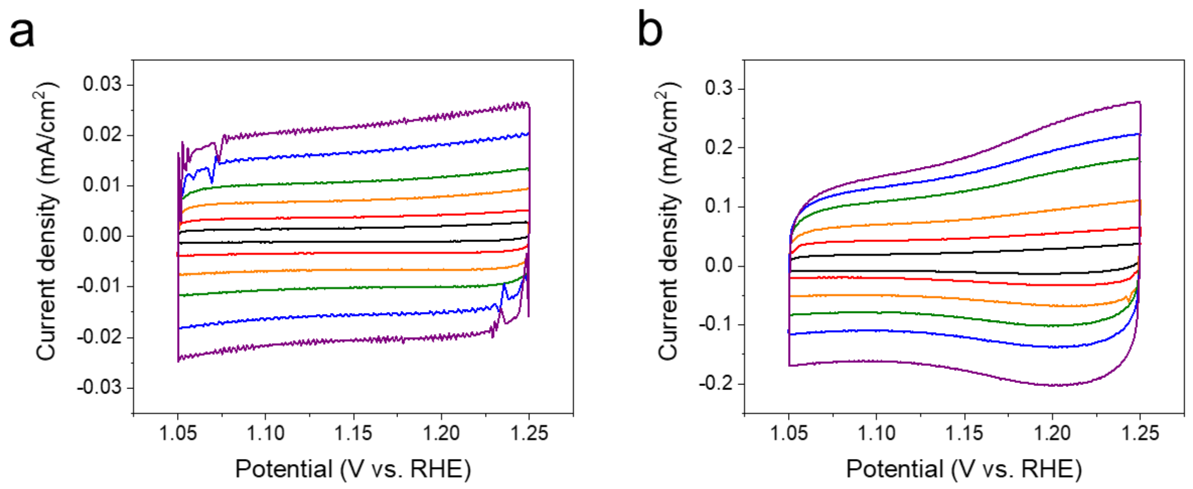

Figure S5. CV curves of a) Ir/Ni film and b) Ir/Ni PNTs with a scan rate of 10 (black), 20 (red), 40 (orange), 60 (olive), 80 (blue), 100 (violet) mV/s.

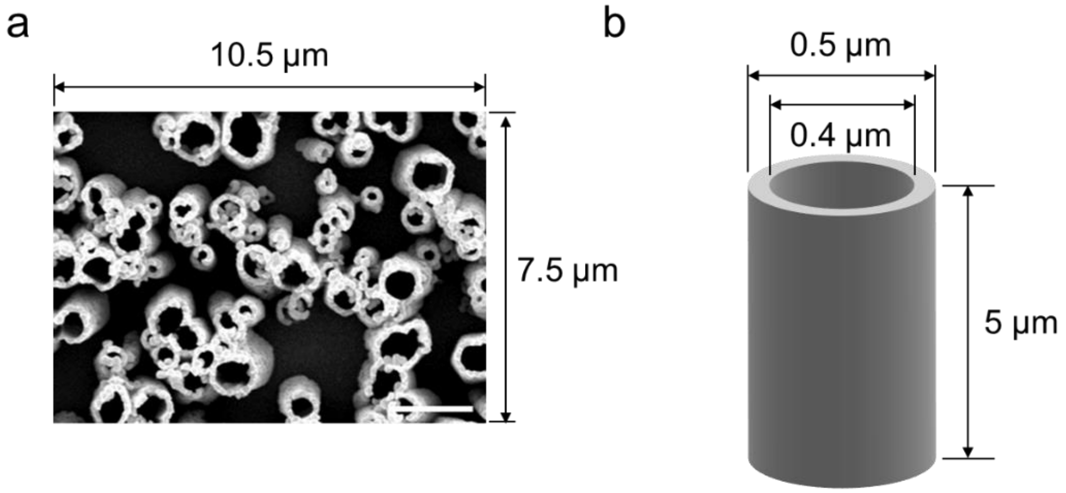

Figure S6. (a) Representative SEM image of PNTs. (b) Schematic of a PNT and estimation of total surface area per a unit projected area.

Note on the calculation of a total surface area per unit area:

$$\text{Number of nanotubes per unit area } (nanotubes/\text{cm}^2) = \frac{60\ nanotubes}{10.5\ \mu\text{m} \times 7.5\ \mu\text{m}}$$
$$= 0.76\, nanotubes/\mu\text{m}^2$$
$$= 7.6 \times 10^{11}\ nanotubes/\text{cm}^2$$

$$\text{Surface area of each nanotube } (\text{cm}^2) = (\text{inner surface area}) + (\text{outer surface area})$$
$$= (0.5\ \mu\text{m} \times \pi \times 5\ \mu\text{m}) + (0.4\ \mu\text{m} \times \pi \times 5\ \mu\text{m})$$
$$= 14.14\ \mu\text{m}^2\ \ = 1.414 \times 10^{-11}\ \text{cm}^2$$

$$\text{Total surface area per unit area } (\text{cm}^2/\text{cm}^2)$$
$$= (7.6 \times 10^{11}\ nanotubes/\text{cm}^2) \times 1.414 \times 10^{-11}\ \text{cm}^2$$
$$= 10.75\ \ \text{cm}^2/\text{cm}^2$$

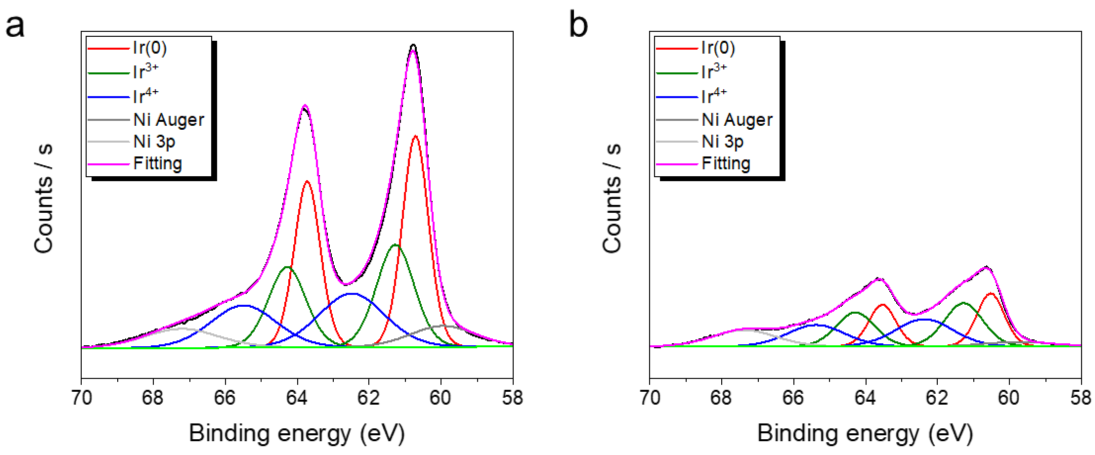

Figure S7. Ir4f XPS spectrum fitting curve of Ir/Ni catalyst a)before and b) after reaction.

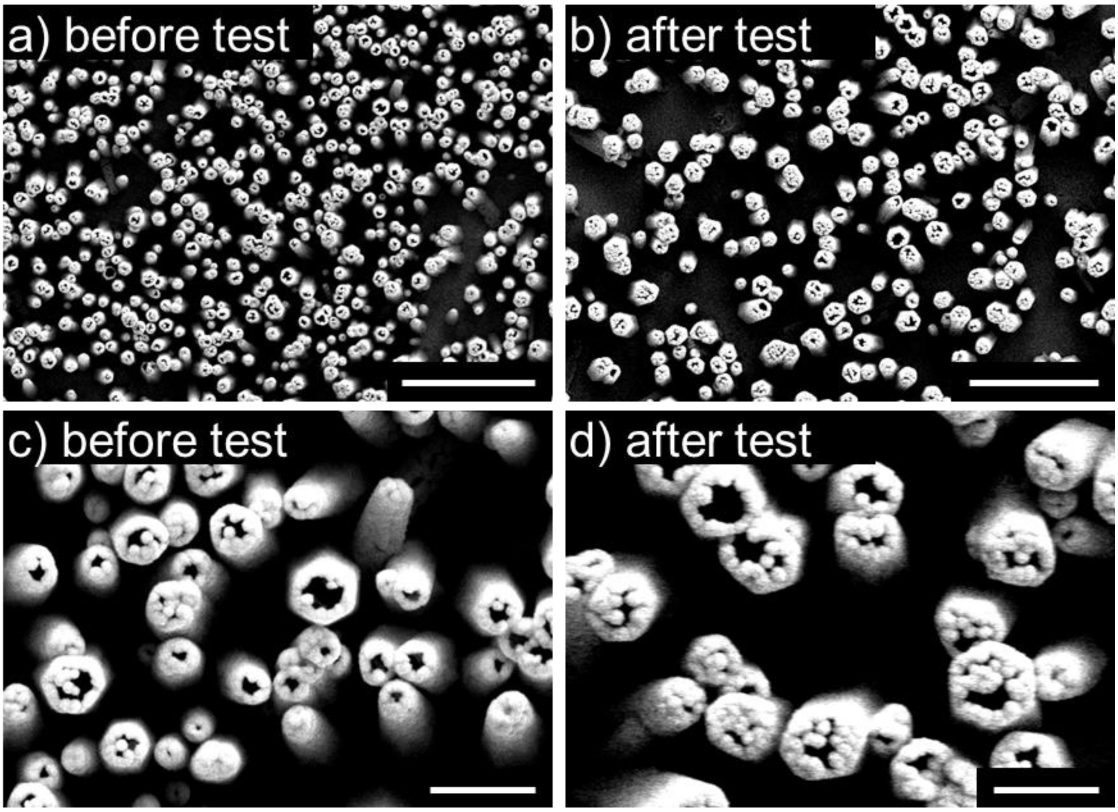

Figure S8. SEM images of Ir/Ni PNTs a, c) before and b, d) after 10 h of OER. The scale bars in a) and b) are 10 μm and those in c) and d) are 2 μm.

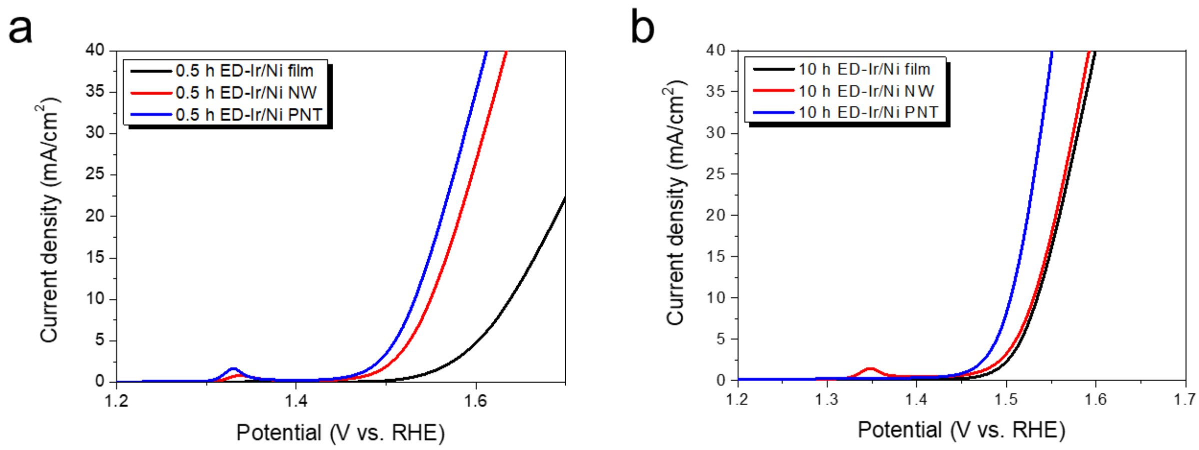

Figure S9. LSV curves of a) 0.5 h ED-Ir/Ni, b) 10 h ED-Ir/Ni catalysts in the structure of planar film, NWs and PNTs.

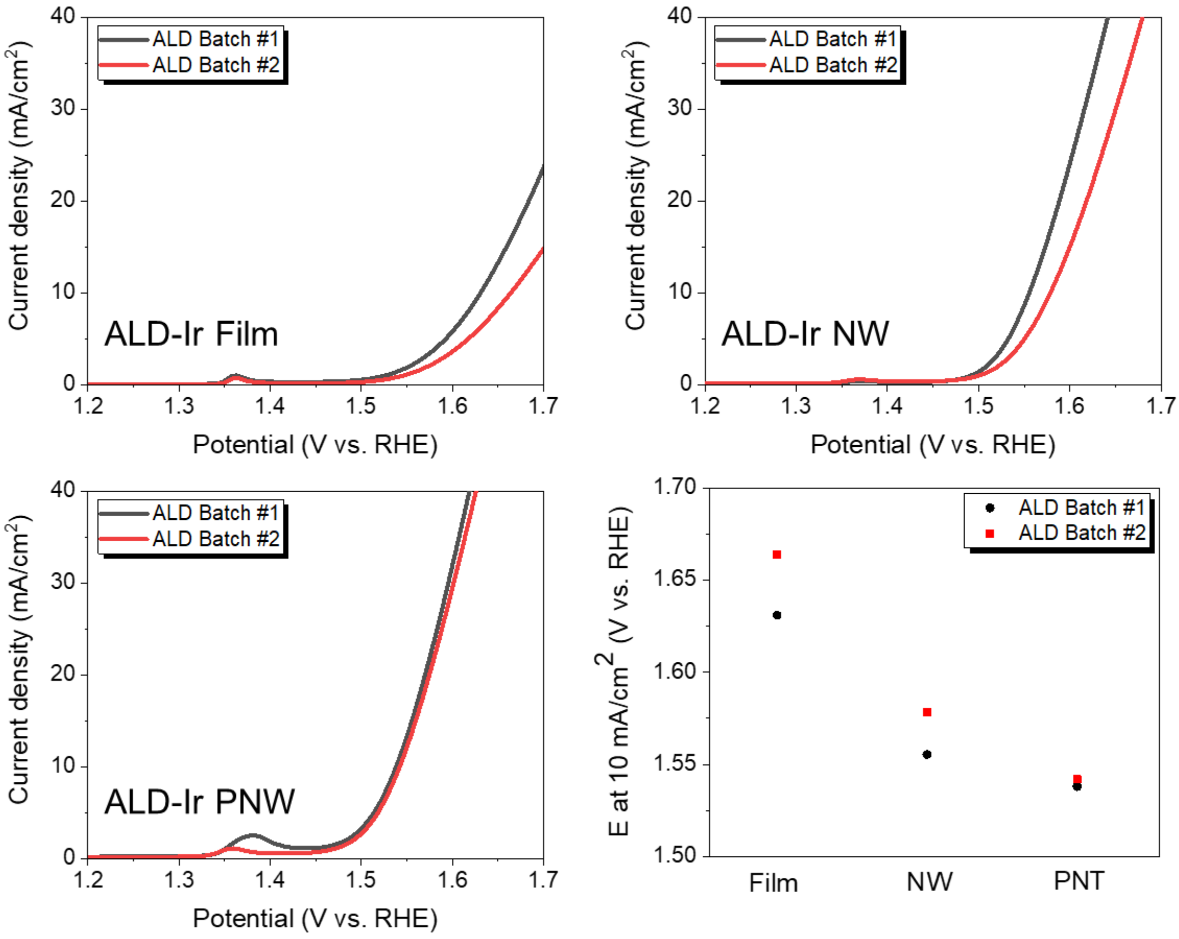

Figure S10. LSV curves and potentials at the operating current density of 10 mA/cm^2 of ALD-Ir/Ni film, NWs, PNTs. The Ir thickness was 2 nm for all the samples.

a

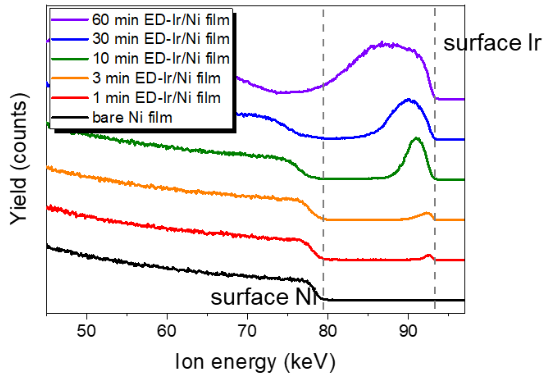

b

Ir_10min						
	Layer	Thick [nm]	Ir	O	C	Ni
	1	1.23	0	0.7134	0.2866	0
	2	0.508	0.08	0.35	0.29	0
	3	0.807	0.23	0.2	0.3	0.07
	4	2.459	0.39	0.16	0.16	0.14
	5	1.293	0.235	0	0	0.765
	6	0.917	0.14	0	0	0.86
	7	4.419	0.011	0	0	0.989
	8	22.025	0	0	0	1
	Total	33.658				
	**IrOx**	**3.774**				
	**Ir-Ni alloy**	**6.629**				
	**Ni**	**∞ (detection limit)**				
	Au	∞ (detection limit)				

c

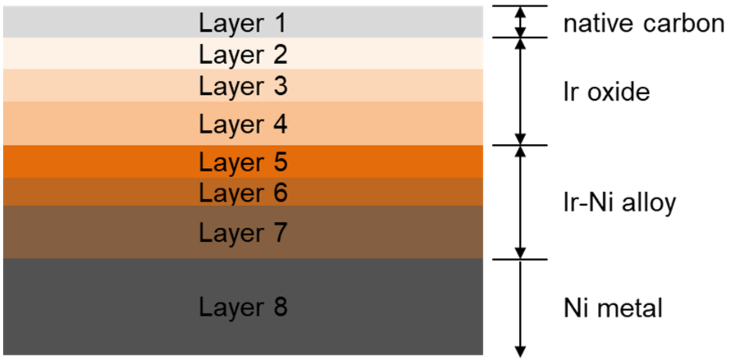

Figure S11. a) MEIS spectra of Ir/Ni films with Ir deposited by ED for 1, 3, 10, 30 and 60 mins. The spectrum from a bare Ni film is also included. b) Fitting results of the MEIS spectrum from the 10 m ED-Ir/Ni film. c) Schematic illustration of 10 min ED-Ir/Ni film according to the fitting result.

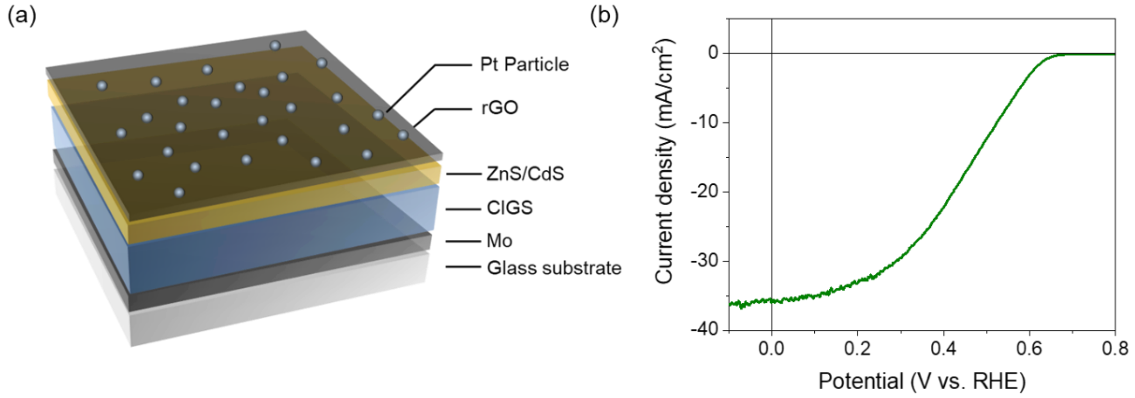

Figure S12. a) Schematic illustration of the device structure and b) LSV curve under 1 sun illumination of CIGS photocathode.

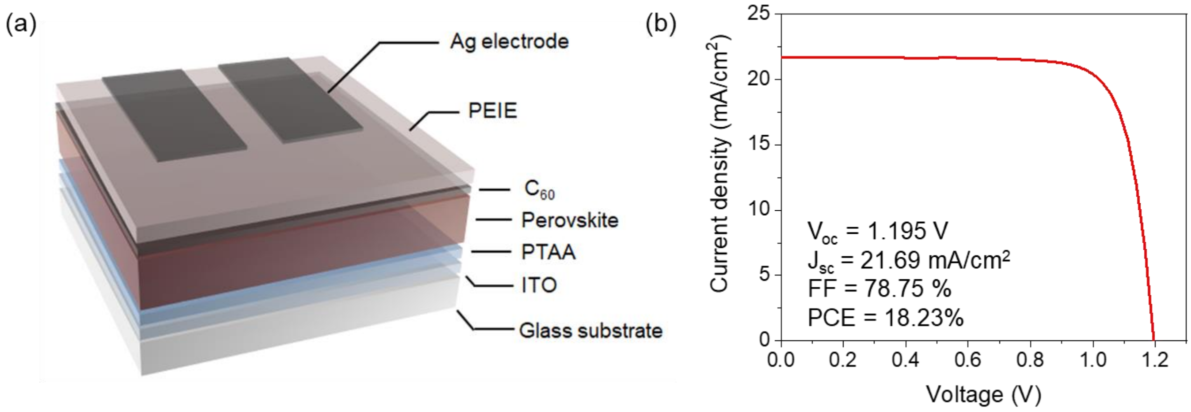

Figure S13. a) Schematic illustration of the device structure and b) current-voltage characteristics of wide bandgap halide perovskite solar cell.

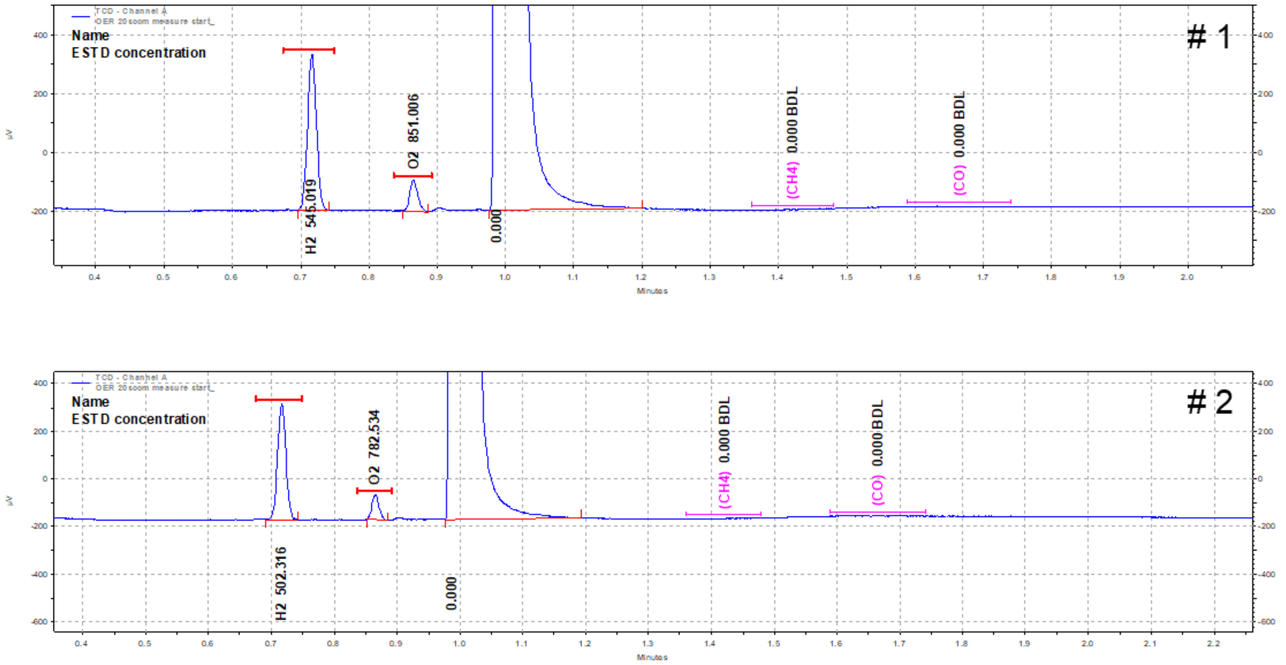

Figure S14. . Gas chromatograph of CIGS photocathode at an operating current of 1.5 mA (13.76 mA/cm2) under 1 sun illumination . A solution consisting of 0.5 M KPi was used as electrolyte.

Calculation of solar-to-hydrogen conversion efficiency

To determine STH efficiency, the tandem PEC-PV system was exposed to the full spectrum of 1 sun illumination of AM 1.5 G. The STH efficiency can be calculated by the following equation:

$$STH\ [\%] = \left[\frac{\left|J_{op}(mA/cm^2)\right| \times (1.23\ V) \times\ \eta_{faradaic}}{P_{total}(mW/cm^2)}\right]_{\mathrm{AM\ 1.5G}} \times 100$$

where J_{op} is the operating current density flowing over the PEC cell in the two-terminal configuration at zero applied bias under 1 sun illumination, the voltage (1.23 V) is the thermodynamically required potential to drive the electrochemical water splitting reaction, $\eta_{faradaic}$ is the Faradaic efficiency which describes the ratio of the experimentally measured amount of evolved hydrogen to the theoretically predicted amount, and the Ptotal indicates the total power density of the incident light. Integrating the AM 1.5 G full spectrum yields a total power density of 100 mW/cm^2, therefore, the STH efficiency equation is reduced to:

$$STH\ [\%] = \left[\left|J_{op}(mA/cm^2)\right| \times (1.23\ V) \times\ \eta_{faradaic}\right]_{\mathrm{AM\ 1.5G}}$$

Note on the calculation of FEs

Charge passed for 1 minutes $= (1.5\ \mathrm{mA}) \times (60\ \mathrm{sec}) = 90\ \mathrm{mC}$

$$mols \text{ of hydrogen produced assuming 100 \% FE} = \frac{90\ \mathrm{mC}}{(96485\ \ \mathrm{C/mol}) \times (2\ \mathrm{electron})}$$

$$= 0.466 \times 10^{-3}\ mmol$$

$$\text{Volume of hydrogen produced} = (22.4\ \mathrm{L/mol}) \times 0.466 \times 10^{-3}\ mmol$$

$$= 0.0104\ m\mathrm{L}$$

Carrier gas flow rate $= 20$ sccm

$$\text{Hydrogen concentration in carrier gas assuming 100 \% FE (ppm)} = \frac{0.0104\ m\mathrm{L}}{20\ m\mathrm{L}} \times 10^{6}$$

$$= 522.361\ \mathrm{ppm}$$

$$\text{FE of measurement \# 1 (\%)} = \frac{545.019\ \mathrm{ppm}}{522.361\ \mathrm{ppm}} \times 100 = 104.3\ \%$$

$$\text{FE of measurement \# 2 (\%)} = \frac{502.316\ \mathrm{ppm}}{522.361\ \mathrm{ppm}} \times 100 = 96.2\ \%$$

The reason for the physically impossible FE over 100% from the first measurement was due to irregular detachment of hydrogen bubbles off the surface of the catalysts.

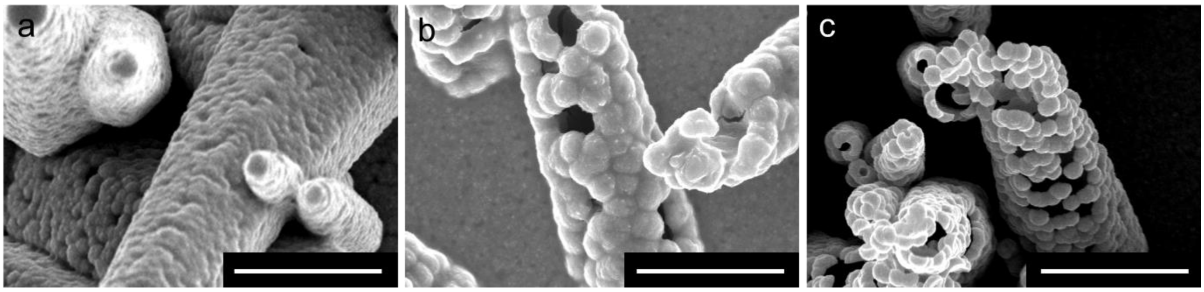

Figure S15. SEM image of Ni PNT with increasing etching duration from (a) to (c). Scale bars are 2 μm.

Film				NW				PNT			
0.5 h	2 h	5 h	10 h	0.5 h	2 h	5 h	10 h	0.5 h	2 h	5 h	10 h
1.62 V	1.625 V	1.615 V	1.608 V	1.567 V	1.607 V	1.55 V	1.566 V	1.516 V	1.55 V	1.526 V	1.504 V
1.621 V	1.611 V	1.6 V	1.529 V	1.533 V	1.567 V	1.526 V	1.526 V	1.59 V	1.557 V	1.531 V	1.516 V
1.653 V	1.538 V	1.534 V	1.514 V	1.553 V	1.612 V	1.605 V	1.53 V	1.508 V	1.533 V	1.526 V	1.512 V

Table S2. Potentials at the operating current density of 10 mA/cm^2 of Ir/Ni film, Ir/Ni NW, Ir/Ni PNT catalysts prepared with 0.5 h, 2 h, 5 h, 10 h of Ir deposition time.

1 min	3 min	10 min	30 min	50 min	60 min	80 min	100 min	120 min
1.71 V	1.63 V	1.588 V	1.62 V	1.586 V	1.623 V	1.556 V	1.552 V	1.595 V
1.663 V	1.655 V	1.565 V	1.621 V		1.584 V			1.602 V
1.67 V	1.66 V	1.589 V	1.61 V		1.635 V			1.572 V
1.689 V	1.678 V	1.62 V	1.618 V		1.623 V			1.585 V
1.667 V	1.633 V	1.537 V	1.608 V		1.564 V			1.563 V
	1.635 V	1.537 V	1.575 V		1.564 V			1.548 V
			1.602 V					
			1.571 V					

Table S3. Potentials at the operating current density of 10 mA/cm^2 of Ir/Ni film catalysts prepared with various Ir deposition times.
